# Preparation and Evaluation of a Calcification Phantom for Mammary Gland MRI

**DOI:** 10.7759/cureus.103315

**Published:** 2026-02-09

**Authors:** Maiko Hashimoto, Norio Hayashi, Masahiko Takahashi

**Affiliations:** 1 Department of Radiology, Isesaki Municipal Hospital, Isesaki, JPN; 2 Department of Radiological Technology, Gunma Prefectural College of Health Sciences Graduate School of Radiological Technology, Maebashi, JPN; 3 Department of Radiological Technology, Gunma Prefectural College of Health Sciences, Maebashi, JPN

**Keywords:** breast mri, calcification, mammography, microcalcification, phantom, quantitative susceptibility mapping

## Abstract

Purpose

Microcalcification detection is vital for early breast cancer diagnosis, treatment, and survival; however, no MRI phantom currently exists for parameter optimization. Therefore, this study aims to develop a breast MRI phantom that simulates calcifications.

Methods

A gelatin-sucrose mixture was prepared, with calcification samples placed on the same plane. MRI using multiple sequences and mammography were performed. Depiction ability was assessed based on contrast, visual evaluation, and statistical analysis.

Results

Among the 0.75-mm samples, eggshell and glass beads showed similar contrast on X-ray and quantitative susceptibility mapping (QSM). While calcifications ≥0.50 mm were identifiable on X-rays, 0.75-mm calcifications were visible on susceptibility-weighted imaging (SWI), T2-weighted imaging (T2WI), and QSM. Scores obtained for eggshell on T1-weighted imaging (T1WI)/T2WI, chicken bone on T1WI/T2WI, and glass beads on SWI were significantly lower than those obtained on X-ray.

Conclusion

A microcalcification phantom enabling X-ray-MRI comparison was developed and validated as useful for MRI-based evaluation of calcifications.

## Introduction

In recent years, breast cancer has become the most common type of cancer among women worldwide [1]. Because breast cancer is highly curable when detected and treated early, timely screening is considered essential [2]. In Japan, mammography screening is recommended every two years as an organized screening program for women aged ≥40 years [3]. However, the cancer detection rate varies for extremely dense breasts, which show a high proportion of glandular tissue on mammographic images. High-risk cases of breast cancer include hereditary breast cancer and dense breasts in younger women, for which mammography has limited effectiveness in detecting masses and calcifications. In addition, some breast cancers can be detected only by MRI [4]. The usefulness of MRI for the diagnosis of breast cancer has been validated in numerous studies [5].

Nonpalpable breast cancer is detected primarily through the presence of microcalcifications during screening [6], accounting for 30-50% of nonpalpable breast cancer cases. The presence of microcalcifications is not only useful as a detection marker but also significant for prognostic evaluation [7].

Several MRI techniques can capture susceptibility differences caused by bleeding and calcification. T2-weighted imaging (T2WI) is an imaging method based on the gradient echo (GRE) technique [8]. T2WI can capture susceptibility differences, such as those caused by bleeding and calcification, as low signal intensities and is commonly used for detecting microbleeds [9]. Susceptibility-weighted imaging (SWI) enhances the contrast of susceptibility differences, allowing more sensitive visualization of minor bleeding and venous malformations than that obtained with T2*WI [10]. Quantitative susceptibility mapping (QSM) enables the quantification of susceptibility variations in biological tissues [11]. The working principle of QSM involves reconstructing the original susceptibility map from the frequency map acquired using techniques such as 3D GRE by solving the inverse problem of dipole kernel convolution [12]. Through the analysis of the obtained phase map and local susceptibility map, QSM can provide material-specific information about hemoglobin, ferritin, calcification, and contrast agents [12]. QSM is particularly useful for distinguishing between diamagnetic substances, such as calcifications (which have negative susceptibility), and paramagnetic substances, such as bleeding (which have positive susceptibility) [11]. Thus, incorporating MRI into breast cancer screening to provide additional calcification information could enhance diagnostic imaging effectiveness.

Several studies have evaluated MRI-based calcification. Wu Z et al. compared SWI phase images with CT for calcification identification [13]. On CT, calcification is considered present if the Hounsfield unit (HU) exceeds a set threshold (100 HU). By contrast, calcification appears with varying signal intensities on conventional spin echo (SE), T1-weighted images (T1WI), or T2WI on MRI, making it difficult to clearly distinguish calcifications [13]. Baheza RA et al. investigated the potential for detecting microcalcifications using 7-T MRI by leveraging the susceptibility difference between calcifications and breast tissue [14]. They reported that with SWI phase images, microcalcifications ranging from 0.6 to 0.9 mm in diameter could be reliably detected within a reasonable scan time [14]. de Leeuw H et al. conducted experiments using breast specimens, with microcalcifications confirmed by mammography, and demonstrated that microcalcifications could be detected using phase imaging [15].

Commercially available phantoms for breast phantom imaging include the Radiation Measurements Inc. (RMI) 156 breast phantom, which is used for quality control in mammography. This phantom is designed with embedded structures mimicking the fibers, calcifications, and masses observed in mammographic imaging. It is used to assess changes in image quality over time and to verify proper system functionality prior to imaging.

MRI breast phantoms include 107 essential breast phantoms designed to evaluate fat suppression, T1 mapping, standardization, spatial resolution, and distortion in quantitative breast MRI. In addition, the PH-72 MRI breast coil evaluation phantom can be used to evaluate MRI performance using breast coils. However, although these MRI phantoms enable quantitative evaluation, they are not designed for calcification assessments.

Phantom-based evaluation is necessary to optimize MRI parameters for the detection of microcalcifications. However, no suitable commercial phantoms are currently available. Given the complexity of breast structures, a simple phantom designed specifically for calcification evaluation is required. Therefore, this study aimed to develop a breast MRI phantom that is effective for assessing calcification-mimicking substances with different primary components.

## Materials and methods

To develop a breast MRI phantom that can enable the accurate evaluation of calcification-mimicking substances with different primary components, three types of microcalcification-mimicking samples (hereafter referred to as “calcification samples”) were used: eggshell, chicken bone, and glass beads of varying sizes. The calcification samples were embedded in gelatin-sucrose gel to ensure their placement within the same imaging plane. The fabricated calcification phantom was imaged using SWI, T2*-WI, QSM, T2WI, and T1WI. For reference, X-ray images of the phantom were acquired using a mammography system.

Equipment and materials

The following materials were used to prepare the phantom: gelatin (Lot LEM6082, FUJIFILM Wako Pure Chemical Corporation, Osaka), sucrose (Lot WTP3011, FUJIFILM Wako Pure Chemical Corporation, Osaka), potassium sorbate (Lot BM-ILKJ-SJ9Y, MARUGO Corporation, Saitama), containers (15 cm × 10 cm), and calcification samples, eggshell, chicken bone, and glass beads (Models BZ-08, BZ-04, and BZ-02, As One Corporation, Osaka). The equipment used included a pure water manufacturing device (PURE LITE PRO-025, Organo Corporation, Tokyo), a microscope (SKYBASIC WiFi Digital Microscope 2MP), a microwave oven (RE-A15KS, Sharp Corporation, Osaka), and an electronic balance (HT-120, A&D Corporation, Tokyo).

The phantom was imaged using an MRI scanner (MAGNETOM Skyra XA30 3T, Siemens Healthcare, Germany) with a 20-channel head-neck receiving coil. It was also imaged using a mammography device (SELENIA Dimensions, Hologic Japan, USA) equipped with a direct-conversion flat-panel X-ray detector with a pixel size of 70 μm. Analyses were performed using QSM analysis software (LiLiby QSM, Fujifilm Healthcare Co., Ltd., Tokyo) and the statistical analysis functions of Prism 5 software (GPW5-113387-RD J-1688; GraphPad Software, San Diego, CA, USA).

Development of breast MRI calcification phantom

The proposed calcification phantom for breast MRI was created in three fundamental steps. First, calcification samples were fabricated from three different materials; then, the background material in which the calcification samples were embedded was prepared; and finally, the calcification phantom was constructed using the calcification samples and background material.

Fabrication of Calcification Samples 

Baheza RA et al., who evaluated calcification on MRI, conducted research on detecting microcalcifications in the breast [14]. In their study, they used 1-mm glass beads as a calcium deposit equivalent. Similarly, Mende J et al. investigated microcalcification detection using eggshells placed on agar to simulate microcalcifications [16]. Calcium phosphate, the main component of bone, is also a main component of calcifications in the breast [17]. Therefore, in this study, microcalcifications were simulated using eggshells, chicken bones, and glass beads as calcification samples.

Calcifications that require differentiation between benign and malignant calcifications are those that cannot be clearly categorized as benign and are judged mainly by their shape and distribution. There are four calcification shapes: (1) microcircular, (2) faint and unclear, (3) polymorphous or heterogeneous, and (4) fine linear or branching. Microcalcifications refer to calcifications measuring 1 mm or less, with those measuring 0.50 mm or less having a higher likelihood of being malignant [18]. Considering these, the diameters of the calcification samples used in this study were set to 0.75 mm, 0.50 mm, and 0.25 mm, based on the diameter of the calcifications embedded in the RMI 156 phantom, which range from 0.54-0.16 mm. The sizes and SDs of the calcification samples are listed in Table 1.

**Table 1:** Sizes and SDs of calcification samples. The number of samples for each material was as follows: eggshells (n = 6), chicken bones (n = 6), and glass beads (n = 6).

Sample type	0.75 mm diam.	0.50 mm diam.	0.25 mm diam.
Eggshell	0.77 ± 0.01	0.49 ± 0.03	0.25 ± 0.02
Chicken bone	0.75 ± 0.04	0.50 ± 0.01	0.25 ± 0.02
Glass beads	0.74 ± 0.04	0.50 ± 0.03	0.25 ± 0.02

The glass beads used were of known diameter, ranging from 0.71-0.99 mm (hereafter referred to as 0.75 mm), 0.30-0.50 mm (hereafter referred to as 0.50 mm), and 0.18-0.25 mm (hereafter referred to as 0.25 mm); the same size ranges were used for the eggshells and chicken bones.

Preparation of Background Medium 

For the background medium, this study referred to a previously developed brain tissue-equivalent phantom used for evaluating diffusion-weighted imaging conditions in acute stroke imaging [19]. A gelatin-sucrose mixture was prepared at a concentration of 12.5 wt%. The preparation involved dissolving 250 g of ultrapure water, 41.7 g of gelatin, and 41.7 g of sucrose in a 500-ml beaker, stirring the mixture, and sealing it with plastic wrap to prevent evaporation. The mixture was heated in a microwave oven at 500 W for 90 s and stirred using a spatula every 15 mins. If the temperature decreased below 40 °C, additional heating was applied for 60 s in a microwave oven.

Construction of Breast MRI Calcification Phantom 

**Figure 1:**
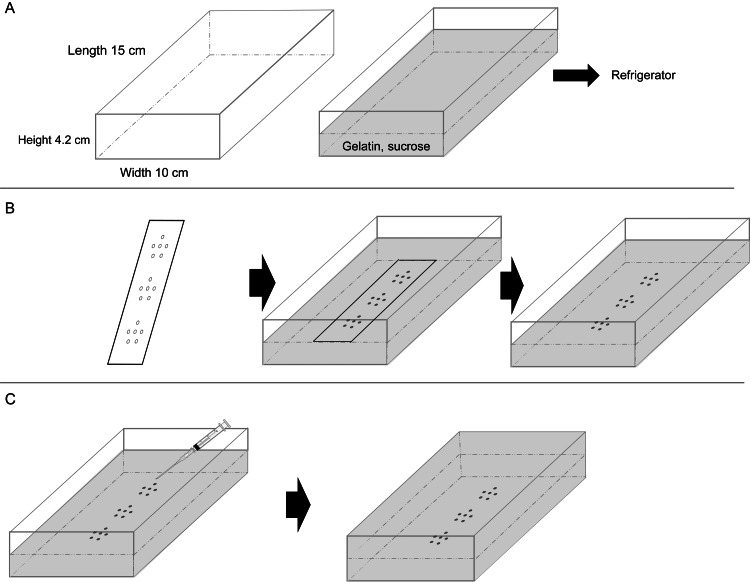
Phantom fabrication procedure. (A) A gelatin-sucrose mixed solution is placed in a plastic container and refrigerated until gelation. (B) A transparent acrylic sheet with six holes (approximately 1 mm in diameter each) is placed in the upper, middle, and lower sections of the gelatinous phantom to position the calcification samples in the same plane. Calcification samples are arranged in the upper, middle, and lower rows. The acrylic sheet is removed after the samples have been placed. (C) The mixed solution is dropped onto the calcification area using a disposable syringe to fix the samples. The remaining mixed solution is then poured into the container. Image credit: Created by the authors.

The procedure for constructing the phantom is illustrated in Figure 1. A mixture of gelatin and sucrose was placed in four plastic containers and allowed to gel in a refrigerator (Figure 1A). A calcification sample was placed in each of the four mixture gels to mimic an RMI 156 phantom. For the first phantom, a transparent acrylic sheet with six holes, each approximately 1 mm in diameter, was placed in the gelatinous phantom at three locations (upper, middle, and lower) to position the calcification samples in the same plane. Subsequently, 0.75-mm-diameter eggshells, chicken bones, and glass beads were placed in the upper, middle, and lower sections of the phantom. For the second phantom, 0.75-mm-, 0.50-mm-, and 0.25-mm-diameter eggshells were placed in the upper, middle, and lower sections. For the third and fourth phantoms, the geometric arrangements were identical to those of the second phantom; however, the materials used for the calcification samples were chicken bones and glass beads (Figure 1B). After the calcification samples were placed, the acrylic sheet was removed. The calcification samples were then fixed by dropping the mixed gelatin-sucrose solution onto them using a disposable syringe. Once the samples were fixed, the remainder of the mixed solution was gradually added to the mixture gel, which was then allowed to set in a refrigerator without any air bubbles (Figure 1C).

Imaging of Breast MRI Calcification Phantom

MRI and X-ray images were acquired to analyze calcifications in the phantom. The phantom was placed at the center of the head-neck coil. The MRI sequences included T2*WI, SWI, and QSM to assess susceptibility differences, alongside the clinically used T1WI and T2WI sequences. The imaging parameters are listed in Table 2.

**Table 2:** MRI and mammography scan parameters. SWI: Susceptibility-weighted imaging; T2*WI: T2*-weighted imaging; QSM: Quantitative susceptibility mapping; T2WI: T2-weighted imaging; T1WI: T1-weighted imaging.

	SWI	T2*WI	QSM	T2WI	T1WI
Matrix	512 × 512	512 × 512	512 × 512	512 × 512	512 × 512
Slice thickness (mm)	2	2	2	2	2
Field of view (mm)	220	220	220	220	220
Echo time (ms)	9.3	12	7.4	81	11
Repetition time (ms)	16	500	13	3200	500
Flip angle (deg)	15	20	20	150	128
Bandwidth (Hz/pix)	200	200	210	199	199
Imaging time (min:s)	1:42	4:18	3:02	2:37	4:18

MRI settings were standardized with a 512 × 512 matrix, 2-mm slice thickness, and a 220-mm field of view (FOV), resulting in a voxel size of 0.4 mm × 0.4 mm × 2.0 mm. SWI and QSM were acquired using 3D sequences, with no post-processing applied to SWI (e.g., minimum intensity projection).

For reference, X-ray images were obtained using an automatic exposure control function on a mammography system with tube voltage settings of 28-30 kV and tube current-time product settings of 112-200 mAs.

Evaluation methods

The fabricated calcification phantom was evaluated based on contrast, visual assessment, and statistical analysis.

*Contrast Measurement* 

Regions of interest (ROIs) of four pixels were set within the calcification sample areas and the adjacent gelatin-sucrose regions. ROIs in the gelatin-sucrose area were positioned such that artifact effects were avoided.

Signal intensity (SI) was measured, and contrast (C) was calculated using the following equation [20]:

C = (SIa − SIb) / (SIa + SIb) …………………………………………………… (1)

where SIa and SIb denote the signal intensities of the calcification samples and the gelatin-sucrose region, respectively.

Visual Assessment 

For the visual assessment, an observer experiment was performed by five observers who are radiological technologists with >10 years of experience. The observers evaluated the phantom based on images obtained for the six calcification samples; the images were displayed on an LCD-MF244EDSW (I-O DATA) monitor adjusted to a brightness of 200 cd/m². The observers visually evaluated the images on a five-point scale. The evaluation criteria were as follows: a score of 5 was given when the sample could be identified as clearly as in mammography; 4, when it could be identified but not as clearly as in mammography; 3, when it could be moderately identified compared with mammography; 2, when it was barely identifiable; and 1, when it was not identifiable. The observer experiment was conducted only after approval had been obtained from the Ethics Committee of the Department of Radiological Technology, Graduate School of Radiological Technology, Gunma Prefectural College of Health Sciences. All observers were provided with sufficient explanations regarding the experiment, and informed consent was obtained in advance. The observers were provided with sufficient training for the experiment. First, the X-ray images were evaluated, and then the calcification samples were evaluated individually, starting with the largest-diameter samples. The averages for the six samples were calculated for each size and material. The visualizability (i.e., the capability to be visualized) of each calcification sample in each sequence and in the created phantom was evaluated based on the results of the visual evaluation.

Statistical analysis

Because the 0.50-mm and 0.25-mm calcification samples were not visually detected, statistical analysis focused on the 0.75-mm samples. The Friedman test, a non-parametric repeated-measures ANOVA, was performed. If significant differences were found, Dunn’s multiple comparison test was used. The significance level was set to P < 0.05. Statistical analyses were performed using Prism 5 software (GraphPad Software, San Diego, CA, USA).

## Results

MRI and X-ray images of calcification phantom

**Figure 2:**
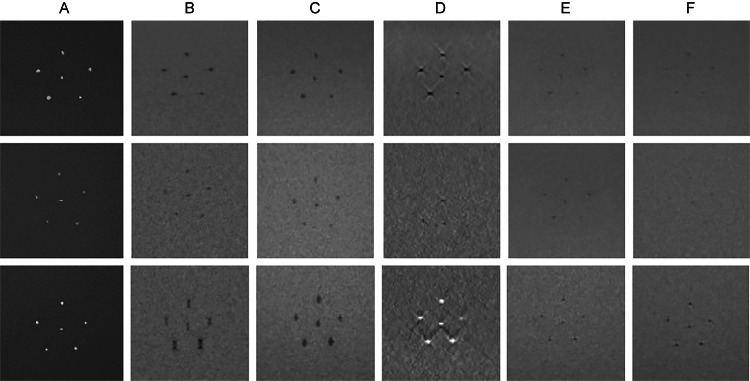
Images of 0.75-mm-diameter calcification phantoms. The images show phantoms containing 0.75-mm-diameter eggshell, chicken bone, and glass bead samples. From top to bottom: eggshell, chicken bone, and glass bead. From left to right: (A) X-ray, (B) SWI, (C) T2*WI, (D) QSM, (E) T2WI, and (F) T1WI. SWI: Susceptibility-weighted imaging; T2*WI: T2*-weighted imaging; QSM: Quantitative susceptibility mapping; T2WI: T2-weighted imaging; T1WI: T1-weighted imaging.

MRI and X-ray images of 0.75-mm eggshell, chicken bone, and glass bead samples are shown in Figure 2. X-ray imaging visualized the eggshells and glass beads clearly, whereas the chicken bones were slightly less distinct but still visible. SWI depicted the eggshells clearly; however, the chicken bones appeared less distinct, and the glass beads appeared elongated along the vertical axis. T2*WI exhibited relatively well-defined visualizations of all calcification samples. QSM demonstrated low signal intensity for the eggshells and chicken bones but high signal intensity for the glass beads. T2WI and T1WI provided indistinct visualizations of all samples.

Contrast measurement results

The contrast values for the 0.75-mm eggshell, chicken bone, and glass bead samples are summarized as follows. In the X-ray images, the contrast values were 0.50 ± 0.02 for eggshells and 0.52 ± 0.01 for glass beads, whereas chicken bone showed a lower contrast of 0.33 ± 0.05. Among the MRI sequences, the contrast values most comparable to those of the X-ray images were observed in QSM: 0.33 ± 0.10 for eggshells and 0.31 ± 0.11 for glass beads. The next closest contrasts were observed in SWI: 0.31 ± 0.14 for glass beads and 0.26 ± 0.09 for eggshells. In T2*WI, the contrast for glass beads was 0.25 ± 0.05.

Table 3 shows the contrast values for the different calcification sample sizes, where A, B, and C indicate eggshell, chicken bone, and glass beads, respectively. For X-ray images of the 0.75-mm-diameter samples, the contrast values were 0.47 ± 0.02 for eggshells and 0.59 ± 0.02 for glass beads, whereas the contrast for chicken bone was slightly lower, at 0.37 ± 0.03. For the 0.50-mm samples, the contrast values were 0.38 ± 0.10 for eggshells, 0.30 ± 0.05 for chicken bone, and 0.26 ± 0.10 for glass beads. In the MRI images, calcification samples ≤0.50 mm exhibited contrast values that were all below 0.16 on average.

**Table 3:** Contrast for simulated calcifications of different sizes. Materials A, B, and C represent eggshell, chicken bone, and glass beads, respectively. SWI: Susceptibility-weighted imaging; T2*WI: T2*-weighted imaging; QSM: Quantitative susceptibility mapping; T2WI: T2-weighted imaging; T1WI: T1-weighted imaging.

Material	Diameter	X-ray	SWI	T2*WI	QSM	T2WI	T1WI
A	0.75 mm	0.47 ± 0.02	0.22 ± 0.05	0.13 ± 0.03	0.27 ± 0.04	0.06 ± 0.02	0.06 ± 0.02
A	0.50 mm	0.38 ± 0.10	0.06 ± 0.05	0.07 ± 0.06	0.07 ± 0.03	0.05 ± 0.03	0.03 ± 0.02
A	0.25 mm	0.01 ± 0.01	0.02 ± 0.02	0.05 ± 0.01	0.04 ± 0.03	0.01 ± 0.01	0.01 ± 0.01
B	0.75 mm	0.37 ± 0.03	0.37 ± 0.08	0.08 ± 0.02	0.40 ± 0.16	0.03 ± 0.01	0.02 ± 0.01
B	0.50 mm	0.30 ± 0.05	0.14 ± 0.08	0.05 ± 0.01	0.15 ± 0.06	0.02 ± 0.01	0.02 ± 0.02
B	0.25 mm	0.05 ± 0.04	0.04 ± 0.02	0.04 ± 0.02	0.04 ± 0.03	0.02 ± 0.01	0.01 ± 0.01
C	0.75 mm	0.59 ± 0.02	0.30 ± 0.06	0.28 ± 0.07	0.44 ± 0.10	0.14 ± 0.03	0.12 ± 0.02
C	0.50 mm	0.26 ± 0.10	0.07 ± 0.03	0.07 ± 0.02	0.16 ± 0.06	0.02 ± 0.02	0.03 ± 0.02
C	0.25 mm	0.04 ± 0.01	0.05 ± 0.03	0.05 ± 0.01	0.08 ± 0.05	0.01 ± 0.01	0.01 ± 0.01

Visual assessment

The results of the visual assessment of the 0.75-mm-diameter calcification samples are as follows.

For the X-ray images, eggshells and glass beads were rated at 5 points, whereas chicken bone scored slightly lower at 4.6 ± 0.5 but was still well distinguishable. SWI and T2*WI also provided clear distinctions. QSM produced good visualizations for eggshells and glass beads but performed poorly for chicken bone, which yielded an average score of only 2.07 ± 0.78. On T2WI, eggshells and glass beads were distinguishable; however, chicken bone was difficult to identify and scored only 2.77 ± 0.43 points. On T1WI, trends similar to those for T2WI were observed: eggshells and glass beads were distinguishable, whereas chicken bone was less discernible and scored lower at 2.37 ± 0.49 points.

Table 4 presents the visual assessment results for the different calcification sample sizes, where A, B, and C indicate eggshell, chicken bone, and glass beads, respectively. For the 0.75-mm-diameter samples, all three materials were generally distinguishable; however, chicken bone exhibited a low score of 2.63 ± 0.56 on T2WI and was completely indistinguishable on T1WI (1 point). For the 0.50-mm-diameter samples, the highest score was obtained for glass beads visualized in QSM, at 2.7 ± 1.02, indicating that they were somewhat distinguishable. For the 0.25-mm samples, the glass beads were barely distinguishable in the X-ray images, yielding a score of only 2.0 ± 0.74, whereas the other materials were not distinguishable even on MRI.

**Table 4:** Visual evaluation of simulated calcifications of different sizes. Materials A, B, and C represent eggshell, chicken bone, and glass beads, respectively. The evaluation criteria were defined as follows: 1 = not identifiable, 2 = barely identifiable, 3 = moderately identifiable compared with mammography, 4 = identifiable but not as clearly as in mammography, and 5 = identifiable as clearly as in mammography. SWI: Susceptibility-weighted imaging; T2*WI: T2*-weighted imaging; QSM: Quantitative susceptibility mapping; T2WI: T2-weighted imaging; T1WI: T1-weighted imaging.

Material	Diameter	X-ray	SWI	T2*WI	QSM	T2WI	T1WI
A	0.75 mm	5.00 ± 0.00	4.80 ± 0.41	5.00 ± 0.00	4.57 ± 0.90	3.87 ± 0.57	4.00 ± 0.74
A	0.50 mm	4.60 ± 0.62	1.77 ± 1.04	2.47 ± 1.20	1.00 ± 0.00	1.13 ± 0.43	1.03 ± 0.18
A	0.25 mm	1.00 ± 0.00	1.00 ± 0.00	1.00 ± 0.00	1.00 ± 0.00	1.00 ± 0.00	1.00 ± 0.00
B	0.75 mm	5.00 ± 0.00	4.80 ± 0.41	3.80 ± 0.76	4.57 ± 0.57	2.63 ± 0.56	1.00 ± 0.00
B	0.50 mm	4.60 ± 0.50	2.50 ± 1.31	2.33 ± 1.09	1.47 ± 0.82	1.00 ± 0.00	1.00 ± 0.00
B	0.25 mm	1.00 ± 0.00	1.03 ± 0.18	1.03 ± 0.18	1.00 ± 0.00	1.00 ± 0.00	1.00 ± 0.00
C	0.75 mm	5.00 ± 0.00	4.00 ± 0.00	5.00 ± 0.00	4.80 ± 0.41	4.80 ± 0.41	4.50 ± 0.57
C	0.50 mm	4.40 ± 0.50	1.97 ± 0.85	2.20 ± 0.85	2.70 ± 1.02	1.10 ± 0.31	1.00 ± 0.00
C	0.25 mm	2.00 ± 0.74	1.10 ± 0.31	1.03 ± 0.18	1.10 ± 0.31	1.00 ± 0.00	1.00 ± 0.00

Statistical analysis

Table 5 shows the results of the statistical analysis of the visual assessment scores for 0.75-mm-diameter samples of eggshells, chicken bones, and glass beads. The Friedman test revealed significant differences among the scores for eggshells, chicken bones, and glass beads (p < 0.0001). Multiple comparisons were performed between X-ray images and the SWI, T2*WI, QSM, T2WI, and T1WI sequences. For eggshells, the scores obtained with T2WI and T1WI were significantly lower than those obtained with X-ray imaging. For chicken bones, the scores obtained with T2WI, T2WI, and T1WI were significantly lower than those obtained with X-ray imaging. For glass beads, the scores obtained with SWI were significantly lower, and the score obtained with T1WI also showed a statistically significant difference.

**Table 5:** Statistical analysis results of visual assessment scores for 0.75-mm-diameter samples of eggshells, chicken bones, and glass beads. ***p < 0.001 (highly significant) and *p < 0.05 (significant), compared with X-ray images. Statistical analysis was performed using the Friedman test followed by Dunn’s multiple comparison test. SWI: Susceptibility-weighted imaging; T2*WI: T2*-weighted imaging; QSM: Quantitative susceptibility mapping; T2WI: T2-weighted imaging; T1WI: T1-weighted imaging.

Material	X-ray	SWI	T2*WI	QSM	T2WI	T1WI
Eggshell	5.00 ± 0.00	4.80 ± 0.41	5.00 ± 0.00	4.57 ± 0.90	3.87 ± 0.57***	4.00 ± 0.74***
Chicken bone	5.00 ± 0.00	4.80 ± 0.41	3.80 ± 0.76***	4.57 ± 0.57	2.63 ± 0.56***	1.00 ± 0.00***
Glass bead	5.00 ± 0.00	4.00 ± 0.00***	5.00 ± 0.00	4.80 ± 0.41	4.80 ± 0.41	4.50 ± 0.57*

## Discussion

In this study, an MRI calcification phantom was successfully developed by mimicking the RMI 156 phantom, enabling the identification of calcification samples even on X-ray imaging. The reference X-ray images confirmed differences in calcification size and placement. Additionally, embedding various calcification sizes revealed that a diameter of 0.25 mm was near the detection limit of X-ray imaging. However, although the 0.25-mm samples were not distinguishable in the contrast measurements or observer experiments, higher-resolution imaging may improve detectability in future applications.

The proposed phantom allows flexible adjustments in calcification size, making it possible to create versions with larger calcifications or intermediate sizes (e.g., 0.40 mm). For the 0.50-mm samples, some were identifiable in the observer experiments, suggesting that altering the slice thickness could improve detectability. The phantom allows different calcification sizes to be embedded in a single imaging plane, enabling evaluation of the minimum detectable size.

For the 0.75-mm calcification samples embedded in the same plane, QSM showed high signal intensity for glass beads, suggesting a compositional difference from human calcifications. This may be attributed to the soda glass material used in the glass beads, which typically contains 0.08%-0.1% iron oxide [21], leading to increased magnetic susceptibility. Consequently, the glass beads exhibited exaggerated susceptibility effects in SWI and T2*WI, making them unsuitable as calcification mimics in QSM. However, given that glass beads have known sizes and reproducibility, they could be useful for T2WI and T1WI assessment. Additionally, in QSM, glass beads may be useful for simulating microbleeds instead of calcifications.

Eggshell samples were clearly distinguishable in the observer experiments using SWI, T2WI, and QSM, making them suitable for these sequences. Chicken bone, which is compositionally closer to human calcifications, may also be suitable for SWI, T2WI, and QSM if appropriately processed for size and shape.

Mustafi D et al. successfully differentiated calcium oxalate and calcium hydroxyapatite crystals in an agarose phantom [22]; however, the fabrication process was complex. By contrast, the phantom fabrication method proposed herein allows flexible adjustments in calcification placement, size, and clustering, making it useful for further detectability evaluations. Additionally, combining glass beads with another calcification sample may enable a QSM-based simulation of both bleeding and calcification.

Although this study used a fixed matrix size and slice thickness, optimizing imaging conditions for calcification visualization in breast tissue could further enhance diagnostic performance. In particular, MRI-based calcification evaluation may be more effective than X-ray imaging for dense breast tissue. If MRI could accurately depict calcifications, it would provide a radiation-free alternative unaffected by breast density, potentially improving screening accuracy when combined with diffusion-weighted imaging.

However, despite its promising results, this study had a number of limitations. When the calcification samples were arranged, holes were made in the acrylic sheet to align their positions; however, the holes were only approximately 1 mm in diameter, and there were irregularities in the distances between the calcification samples. This may have caused positional inaccuracies and affected contrast measurements. When the sizes of the three calcification samples embedded inside the phantoms were aligned, the sizes were extracted using a microscope; however, accurate lengths and alignments could not be determined. The thicknesses and three-dimensional structures of the calcification samples may also be inconsistent. This inconsistency may have caused changes in signal intensity owing to partial volume effects during imaging. To address these problems, variability in calcification sample size can be reduced by measuring sample volume and length using a microscope. Each sample with a diameter of 0.75 mm was evaluated using multiple phantoms. The evaluation results exhibited similar trends among the phantoms; however, the numerical values differed, which may be attributed to variations in the calcification samples between the phantoms and errors in the measurement process.

## Conclusions

This study successfully developed an MRI phantom for simulating microcalcifications. The phantom allows calcifications of different sizes to be embedded within the same imaging plane, a feature not observed in previously developed phantoms. It enables direct comparison between X-ray imaging and various MRI sequences, facilitating assessment of calcification visibility and optimization of imaging parameters. Therefore, the phantom is expected to be a valuable tool for MRI-based evaluation of calcifications.
